# Heterosynaptic NMDA receptor plasticity in hippocampal dentate granule cells

**DOI:** 10.3389/fnsyn.2026.1825949

**Published:** 2026-07-22

**Authors:** Kaoutsar Nasrallah, Maryann Castillo, Stefano Lutzu, Pablo E. Castillo, Alma Rodenas-Ruano

**Affiliations:** 1 Dominick P. Purpura Department of Neuroscience, Albert Einstein College of Medicine, Bronx, NY, United States; 2 Department of Biological Sciences, Fordham University, Bronx, NY, United States; 3 Department of Psychiatry and Behavioral Sciences, Albert Einstein College of Medicine, Bronx, NY, United States; 4 Department of Natural Sciences, Fordham University, New York, NY, United States

**Keywords:** AMPA and NMDA receptors, entorhinal cortex, hippocampus, long-term depression (LTD), long-term potentiation (LTP)

## Abstract

The dentate gyrus is a key relay station that controls information transfer from the entorhinal cortex to the hippocampus proper. This process relies heavily on dendritic integration by dentate granule cells (GCs) of excitatory synaptic inputs from the medial and lateral entorhinal cortex via medial and lateral perforant paths (MPP and LPP, respectively). Inputs from the entorhinal cortex onto GCs exhibit activity-dependent long-term plasticity of N-methyl-D-aspartate receptor (NMDAR)-mediated synaptic transmission. However, the properties, underlying mechanisms, and input-specificity of this plasticity remain poorly understood. Here, we examined NMDAR plasticity rules at MPP-GC and LPP-GC synapses using physiologically relevant stimulation patterns in acute hippocampal slices from rats and mice. Unlike MPP-GC synapses, LPP-GC synapses did not express homosynaptic NMDAR-LTP. Additionally, inducing NMDAR-LTP at MPP-GC synapses potentiated NMDAR transmission at distal LPP-GC synapses. The same stimulation protocol induced homosynaptic α-amino-3-hydroxy-5-methyl-4-isoxazolepropionic acid receptor (AMPAR)-LTP at MPP-GC synapses but heterosynaptic AMPAR-LTD at distal LPP synapses, indicating that NMDAR and AMPAR plasticity are controlled by different plasticity rules. Notably, heterosynaptic but not homosynaptic NMDAR-LTP required Ca^2+^ release from intracellular, ryanodine receptor-dependent Ca^2+^ stores. Lastly, genetic deletion of the GluN2D subunit from GCs or selective antagonism of GluN2D-containing NMDARs abolished heterosynaptic LTP. Heterosynaptic NMDAR-mediated LTP may have important consequences for the dendritic integration of functionally distinct excitatory inputs by dentate granule cells.

## Introduction

Activity-dependent changes in synaptic strength are widely regarded as a key mechanism underlying experience-driven refinement of neuronal circuits and memory formation (Mayford et al., 2012, Takeuchi et al., 2014). Synaptic plasticity can manifest in multiple forms, including homo- and heterosynaptic plasticity. Homosynaptic plasticity is expressed only at stimulated synapses, whereas heterosynaptic plasticity involves changes in strength at neighboring unstimulated synapses. Although it is well-established that glutamate NMDA receptors (NMDARs) mediate excitatory synaptic transmission and can undergo long-term potentiation (LTP) and long-term depression (LTD) (Hunt and Castillo, 2012, Rebola et al., 2010), most studies have focused on homosynaptic (Diering and Huganir, 2018, Herring and Nicoll, 2016) and heterosynaptic (Chater and Goda, 2021, Malyshev et al., 2026, Jenks et al., 2021) plasticity of the AMPA receptor (AMPAR)-mediated component of glutamatergic excitatory transmission. However, whether NMDAR-mediated transmission undergoes heterosynaptic plasticity remains to be investigated.

The dentate gyrus is the main input area of the hippocampus (Amaral et al., 2007, Jonas and Lisman, 2014). Dentate gyrus granule cells (GCs) receive prominent cortical projections from the medial and lateral entorhinal cortex, which convey context and content-related information, through the medial and lateral perforant pathways (MPP and LPP), respectively (Knierim et al., 2014). Activity-dependent changes of these inputs, and their dendritic integration by GCs are critical to dentate gyrus information processing (Knierim et al., 2014, Krause et al., 2008, Krueppel et al., 2011, Schmidt-Hieber et al., 2007, Hainmueller and Bartos, 2020). MPP and LPP inputs onto GCs express robust homosynaptic (Bliss and Lomo, 1973, Colino and Malenka, 1993, Richter-Levin et al., 1995) and heterosynaptic (Abraham et al., 2007, Jungenitz et al., 2018) long-term plasticity of AMPAR-mediated transmission. Furthermore, early studies showed that high-frequency stimulation can also trigger homosynaptic NMDAR plasticity in perforant path inputs (Harney et al., 2006, O’Connor et al., 1994). Using physiologically relevant patterns of presynaptic and postsynaptic burst activity, a protocol known as burst-timing-dependent plasticity (BTDP), we have recently shown that MPP-GC synapses can undergo robust NMDAR LTP but not NMDAR-LTD (Berthoux et al., 2026). Whether LPP-GC synapses can express homo- or heterosynaptic NMDAR plasticity is unknown.

Here, we examined whether BTDP induction protocols induce NMDAR plasticity at LPP inputs in acute hippocampal slices from rats and mice. We found that LPP-GC synapses do not express homosynaptic NMDAR plasticity but can undergo heterosynaptic plasticity induced by MPP-GC synapses. Conversely, homosynaptic AMPAR-LTP at MPP-GC synapses was accompanied by heterosynaptic AMPAR-LTD at LPP-GC synapses. Heterosynaptic NMDAR-LTP increased LPP-driven GC firing and was blocked by GluN2D antagonism and *Grin2d* postsynaptic conditional knockout. To our knowledge, these findings provide the first evidence of heterosynaptic LTP of NMDAR-mediated transmission and suggest that BTDP-induced homosynaptic plasticity at MPP can shape the transfer of information from LPP to GCs, thereby contributing to dentate gyrus-dependent forms of memory.

## Materials and methods

### Experimental model and subject details

Postnatal day 19 (P19) to P30 Sprague-Dawley rats (Charles River), P30 to P60 C57BL/6 (Jackson Laboratory) and floxed *Grin2d* mice (*Grin2d ^*fl*/*fl*^*), of either sex were used for electrophysiological experiments. The *Grin2d ^*fl*/*fl*^* mice were generated by removing the reporter cassette by crossing *Grin2d*^*TM*1a(EUCOMM)Wtsi^ mice with B6-SJL-Tg(ACTFLPe)9205Dym/J line. All animals were group-housed under a standard 12 h light/12 h dark cycle. Animal handling and use followed a protocol approved by the Animal Care and Use Committee of Albert Einstein College of Medicine, in accordance with the National Institutes of Health guidelines.

### Hippocampal slice preparation

Acute dorsal and intermediate transverse hippocampal slices (300–400 μm thick) were prepared from Sprague–Dawley rats, C57BL/6 and *Grin2d ^*fl*/*fl*^* mice (300 μm thick). Briefly, the hippocampi were isolated and cut using a VT1200s microslicer (Leica Microsystems Co.) in a solution containing (in mM): 215 sucrose, 2.5 KCl, 26 NaHCO_3_, 1.6 NaH_2_PO_4_, 1 CaCl_2_, 4 MgCl_2_, 4 MgSO_4_ and 20 D-glucose. At 30 min post-sectioning, the cutting medium was gradually switched to extracellular artificial cerebrospinal fluid (ACSF) recording solution containing (in mM): 124 NaCl, 2.5 KCl, 26 NaHCO_3_, 1 NaH_2_PO_4_, 2.5 CaCl_2_, 1.3 MgSO_4_ and 10 D-glucose. Mouse hippocampal slices were prepared using an NMDG-based cutting solution containing (in mM): 93 N-Methyl-*d*-glucamin, 2.5 KCl, 1.25 NaH_2_PO_4_, 30 NaHCO_3_, 20 HEPES, 25 D-glucose, 2 Thiourea, 5 Na-Ascorbate, 3 Na-Pyruvate, 0.5 CaCl_2_, 10 MgCl_2_. The NMDG-based cutting solution has been shown to improve tissue quality when preparing slices from older mice (*P* > 30) (Ting et al., 2014). Slices were incubated in ACSF at room temperature for at least 45 min before recording.

### Electrophysiology

All experiments, unless otherwise stated, were performed at 28 ± 1 °C in a submersion-type recording chamber perfused at ∼2 mL min^–1^ with ACSF. Whole-cell patch-clamp recordings were made from GCs voltage clamped at −45 mV (unless otherwise stated) using a Multiclamp 700A amplifier (Molecular Devices) and patch-type pipette electrodes (∼3–4 MΩ) containing (in mM): 135 K-Gluconate, 5 KCl, 0.1 EGTA, 0.04 CaCl_2_, 5 NaOH, 5 NaCl, 10 HEPES, 5 MgATP, 0.4 Na_3_GTP, and 10 D-glucose, pH 7.2 (280–290 mOsm). Both series resistance (∼7–25 MΩ) and input resistance were monitored throughout all experiments with a −5 mV, 80 ms voltage step to ensure recording quality, and cells showing a significant change in series resistance (>20%) were excluded from analysis.

**FIGURE 1:**
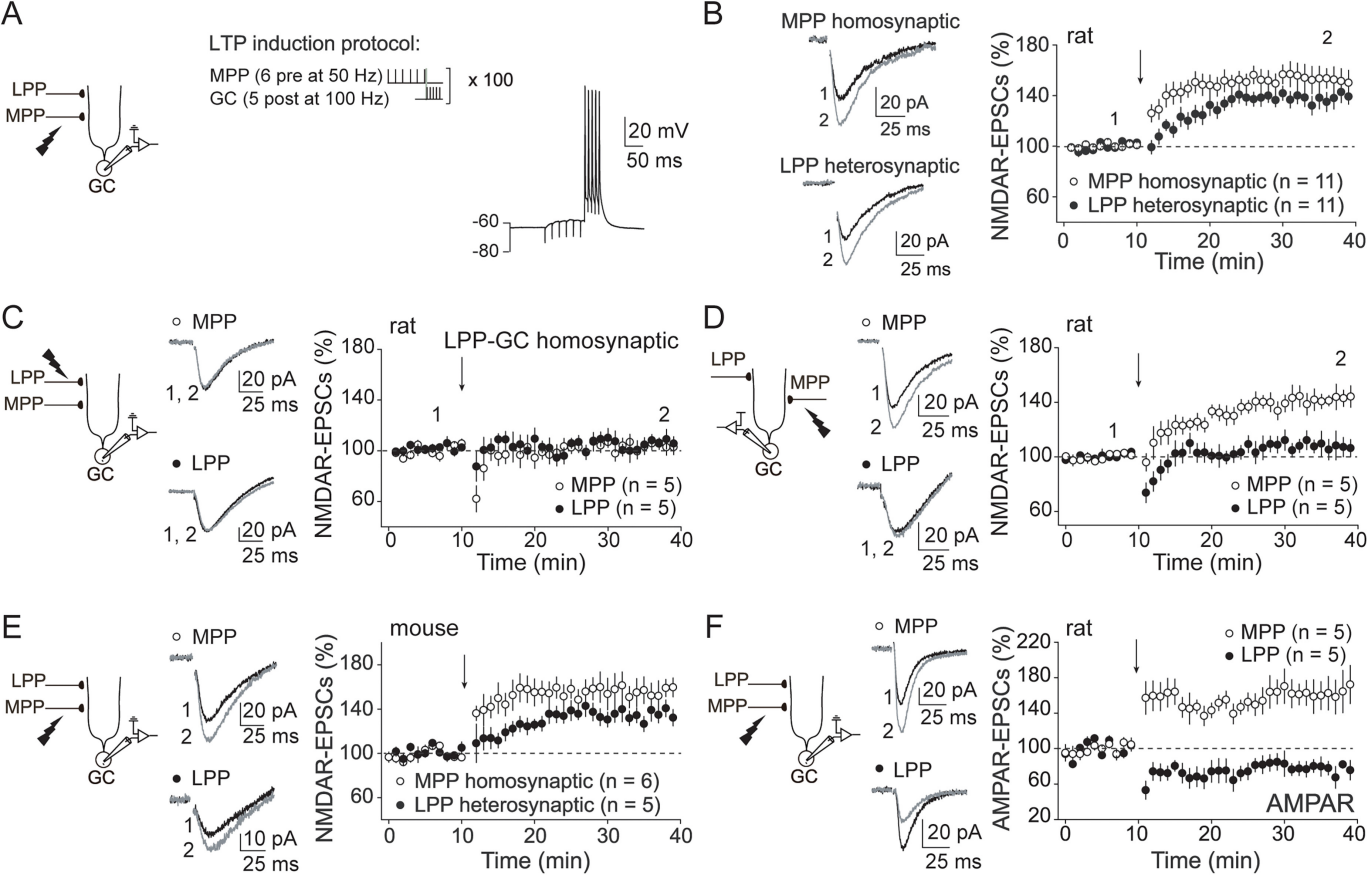
LPP-GC synapses express heterosynaptic NMDAR-LTP but heterosynaptic AMPAR-LTD. **(A)** Diagram illustrating both the recording configuration and the LTP induction protocol. LPP and MPP NMDAR EPSCs were recorded from the same GC. Stimulating electrodes were placed in the most distal border of the outer molecular layer (OML) and in the middle molecular layer (MML) of the dentate gyrus to stimulate LPP and MPP, respectively. The LTP induction protocol consisted of a pre-post pairing protocol applied at MPP (6 pre-pulses at 50 Hz followed by 5 post-pulses at 100 Hz with a 10 ms interval, repeated 100 times every 0.5 s). An example trace of a single pairing from the pre–post protocol is shown on the right. **(B)** Left, Representative average traces before and after LTP induction. Right, Time-course summary plot showing how pre-post pairing protocol induced at MPP (6 pre pulses at 50 Hz followed by 5 post pulses at 100 Hz with a 10 ms interval, repeated 100 times every 0.5 s), resulted in homosynaptic NMDAR-LTP at MPP-GC synapses (white circles, 153.1 ± 7.9 %, *p* < 0.001, *n* = 11, paired *t*-test, g_av_ = 7.4), accompanied by heterosynaptic NMDAR-LTP at distal LPP-GC synapses (black circles, 137.2 ± 5.1%, *p* < 0.001, *n* = 11, paired *t*-test, g_av_ = 3.6). **(C)** Left, diagram illustrating the stimulation and recording configuration. Right, traces (middle) and summary data (right) showing that a pre-post pairing protocol applied at LPP failed to elicit homosynaptic NMDAR LTP at LPP-GC synapses (105.3 ± 4.5%, *p* = 0.30, *n = 5*, paired *t*-test, g_av_ = 0.49) and heterosynaptic NMDAR LTP at MPP-GC synapses (103.6 ± 4.5%, *p* = 0.43, *n* = 5, paired *t*-test, g_av_ = 0.19). **(D)** Left, diagram illustrating the stimulation and recording configuration. MPP and LPP NMDAR EPSCs were recorded from the same GC. The LPP stimulation micropipette was placed on the distal OML, at least 250 μm lateral from the location of the MPP stimulation micropipette. The stimulation pipettes were placed on opposite sides relative to the recorded cell to recruit inputs onto distinct GC dendritic branches. Middle, representative average traces before and after LTP induction. Right, summary plot showing that pre-post pairing elicited homosynaptic LTP at MPP-GC synapses (white circles, 142.6 ± 5.6%, *p* < 0.01, *n = 5*, paired *t*-test, g_av_ = 10.6) while it did not trigger heterosynaptic LTP at LPP-GC synapses (black circles, 105.3 ± 4.5%, *p* = 0.25, *n = 5*, paired *t*-test, g_av_ = 1.9). **(E)** Representative traces and summary data from experiments in acute mouse hippocampal slices reveal that heterosynaptic NMDAR-dependent LTP at dentate granule cells is a conserved phenomenon. The pre-post pairing protocol induced at MPP resulted in homosynaptic NMDAR-LTP at MPP-GC synapses (white circles, 155.8 ± 11.3%, *p* < 0.01, *n* = 6, paired *t*-test, g_av_ = 9.3), accompanied by heterosynaptic LTP at distal LPP-GC synapses (black circles, 134.5 ± 6.8%, *p* < 0.01, *n* = 5, paired *t*-test, g_av_ = 5.5). **(F)** Left, Diagram illustrating the recording configuration. MPP and LPP AMPAR EPSCs were recorded from the same GC. Middle, representative traces. Right, Time-course summary plot shows that pre-post pairing protocol at MPP inputs induced homosynaptic AMPAR-LTP at MPP-GC synapses (white circles, 163.2 ± 15.8%, *p* < 0.05, *n* = 5, paired *t*-test, g_av_ = 7.6), while it triggered heterosynaptic AMPAR-LTD at LPP-GC synapses (black circles, 77.7 ± 7.6%, *p* < 0.05, *n* = 5, paired *t*-test, g_av_ = –3.4). Numbers in parentheses indicate the number of cells. Summary data represent mean ± s.e.m.

**FIGURE 2:**
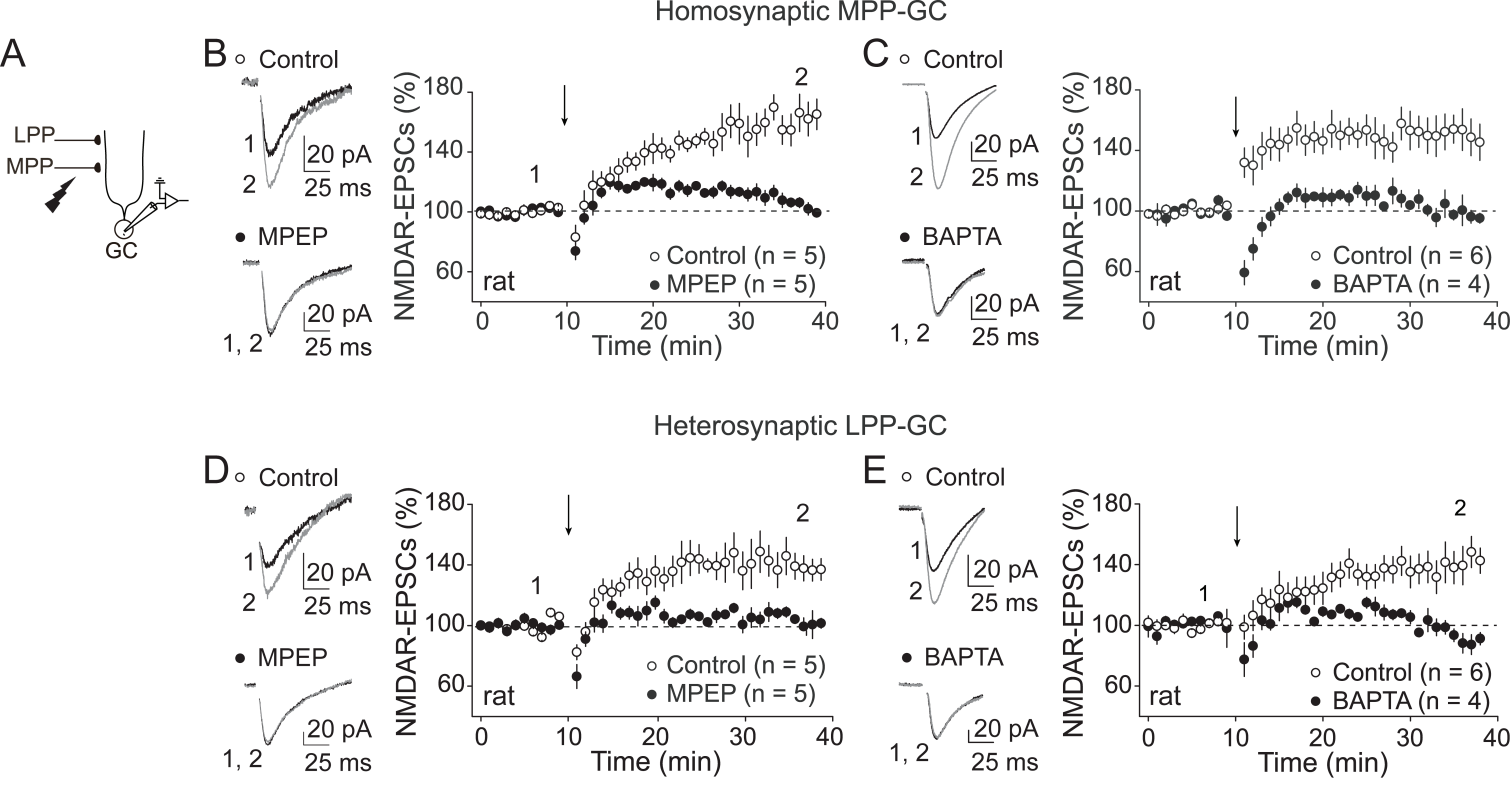
MPEP and BAPTA blocked both homosynaptic and heterosynaptic NMDAR-LTP. **(A–E)** LPP and MPP NMDAR EPSCs were recorded from the same GC, and a pre-post pairing protocol was delivered at MPP inputs. **(B)** Bath application of the mGluR5 antagonist MPEP (4 μM) significantly reduced homosynaptic NMDAR LTP (black circles, 107.6 ± 2.4%, *n* = 5, g_av_ = 0.61) as compared with interleaved controls (white circles, 154.3 ± 9.5%, *p* < 0.01, *n* = 5, g_av_ = 7.43, paired *t*-test; MPEP vs. control: *p* < 0.01, unpaired *t*-test, *g* = –11.68). **(C)** Intracellular loading of the Ca^2+^ chelating agent BAPTA (10 mM) abolished homosynaptic NMDAR-LTP (black circles, 98.9 ± 3.4%, *p* = 0.76, *n* = 4, paired *t*-test, g_av_ = –0.17) relative to controls (white circles, 151.7 ± 11.4%, *p* < 0.01, *n* = 6, paired *t*-test, g_av_ = 6.99; BAPTA vs. control: *p* < 0.01, unpaired *t*-test, *g* = –12.49). **(D,E)** Both MPEP (4 μM) application and intracellular loading of BAPTA (10 mM) impaired heterosynaptic NMDAR-LTP at LPP-GC synapses [**(D)** MPEP: 105.6 ± 3.3%, *p* = 0.16, *n* = 5, paired *t*-test, g_av_ = 0.62, MPEP vs. control: *p* < 0.01, unpaired *t*-test, *g* = –7.63; **(E)** BAPTA: 98.0 ± 1.9%, *p* = 0.36, *n* = 4, paired *t*-test, g_av_ = –0.23; BAPTA vs. control: *p* = 0.02, unpaired *t*-test, *g* = –8.80]. Numbers in parentheses indicate the number of cells. Summary data represent mean ± s.e.m.

**FIGURE 3:**
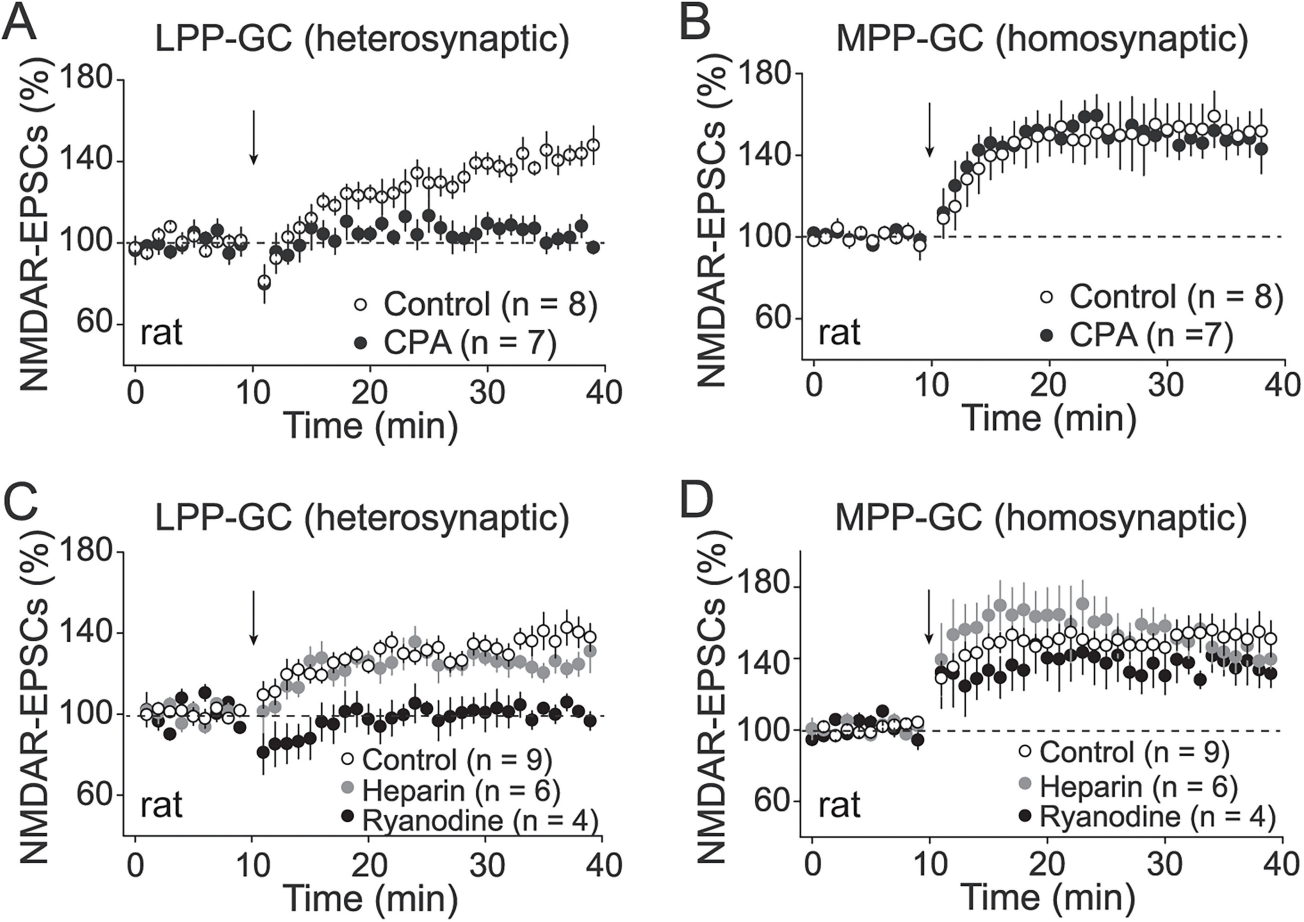
Internal Ca^2+^ stores are required for heterosynaptic but not homosynaptic NMDAR-LTP. **(A)** Application of the sarcoplasmic reticulum Ca^2+^-ATPase inhibitor CPA (30 μM, 40–50 min pre-incubation and bath application) abolished heterosynaptic NMDAR-LTP at LPP-GC synapses (black circles, 106.6 ± 3.8%, *p* = 0.13, *n* = 7, paired *t*-test, g_av_ = 0.52), relative to interleaved controls (white circles, 140.5 ± 4.2%, *p* < 0.001, *n* = 8, paired *t*-test, g_av_ = 3.20, CPA vs. control: *p* < 0.001, unpaired *t*-test, *g* = –6.07). **(B)** In contrast, CPA application had no significant effect on homosynaptic NMDAR-LTP at MPP-GC synapses (black circles, 149.4 ± 6.5%, *p* < 0.001, *n* = 7, paired *t*-test, g_*av*_ = 6.33) as compared with interleaved controls (white circles, 151.3 ± 7.8%, *p* < 0.001, *n* = 8, paired *t*-test, g_*av*_ = 5.43; CPA vs. control: *p* = 0.75, unpaired *t*-test, *g* = –0.38). **(C)** Heterosynaptic plasticity was blocked by ryanodine (100 μM, 40–50 min pre-incubation and bath application) (black circles, 101.4 ± 4.1%, *p* = 0.76, *n* = 4, paired *t*-test, g_av_ = 0.73, ryanodine vs. control: *p* < 0.05, unpaired *t*-test, *g* = –6.95), but was comparable to controls (white circles, 135.4 ± 5.6%, *p* < 0.001, *n* = 9, paired *t*-test, g_av_ = 3.72) in the presence of the IP3 receptor inhibitor heparin (2 mg/ml, 40–50 min pre-incubation and bath application) (gray circles, 124.2 ± 4.0%, *p* < 0.01, *n* = 6, paired *t*-test, g_av_ = 2.74, heparin vs. control: *p* = 0.44, unpaired *t*-test, *g* = –2.39). **(D)** Inhibition of ryanodine (black circles, 135.3 ± 8.7%, *p* < 0.05, *n* = 4, paired *t*-test, g_av_ = 4.40; ryanodine vs. control: *p* = 0.46, unpaired *t*-test) or IP3 (gray circles, 146.5 ± 7.1%, *p* < 0.01, *n* = 6, paired *t*-test, g_av_ = 4.93; heparin vs. control: *p* = 0.44, unpaired *t*-test) receptors had no significant effect on homosynaptic NMDAR-LTP at MPP-GC synapses compared with interleaved controls (white circles, 152.6 ± 8.9%, *p* < 0.001, *n* = 9, paired *t*-test, g_av_ = 6.33). Numbers in parentheses indicate the number of cells. Summary data represent mean ± s.e.m.

**FIGURE 4:**
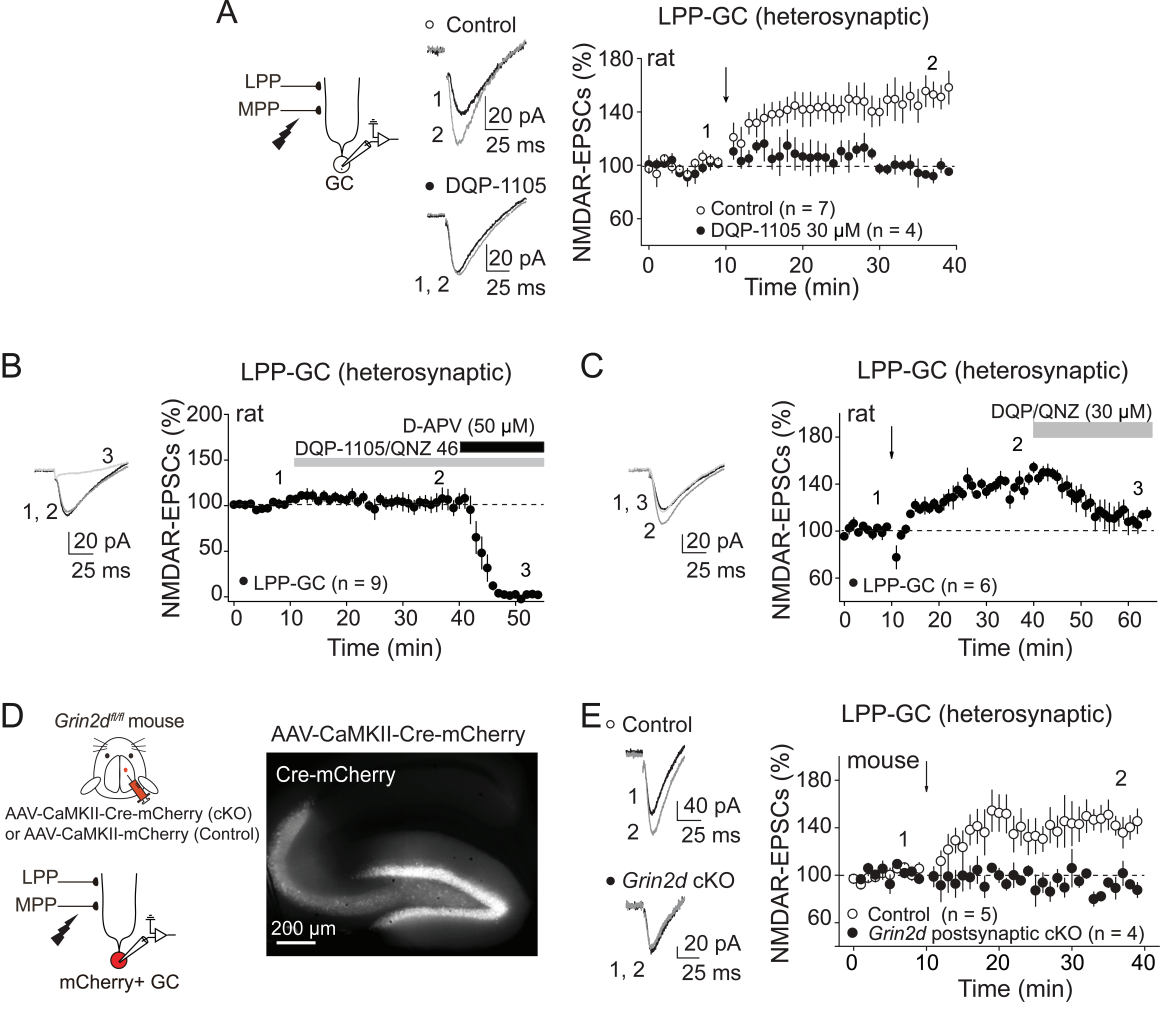
Heterosynaptic NMDAR-LTP is likely mediated by synaptic recruitment of GluN2D-containing receptors. **(A)** NMDAR EPSCs were evoked by LPP stimulation. The pre-post pairing LTP induction protocol was delivered to MPP inputs. Induction of heterosynaptic NMDAR-LTP at LPP-GC synapses was blocked in the presence of 30 μM DQP-1105 as compared to interleaved controls (control: 147.8 ± 9.1%, *p* < 0.01, *n* = 7, paired *t*-test, g_*av*_ = 4.22; DQP-1105: 99.1 ± 2.2%, *p* = 0.71, *n* = 4, paired *t*-test, g_*av*_ = –0.06, DQP-1105 vs. control: *p* < 0.01, unpaired *t*-test, *g* = –9.30). **(B)** Bath application of DQP-1105 or QNZ46 had no effect on basal NMDAR synaptic transmission (103.4 ± 7.7%, *p* = 0.67, *n* = 9, paired *t*-test, g_*av*_ = 0.37). Application of D-APV abolished these EPSCs, confirming that these responses were mediated by NMDARs (LPP: 9.0 ± 3.7%, *p* < 0.001, paired *t*-test). **(C)** Bath application of either DQP-1105 (30 μM) or QNZ46 (30 μM) 30 min after NMDAR-LTP induction substantially reduced (∼85%) heterosynaptic NMDAR-EPSC amplitude (DQP-1105 vs. post-LTP: *p* < 0.05, unpaired *t*-test, g_*av*_ = –2.46). **(D)** Left, postsynaptic *Grin2d* conditional knockouts (cKO) were obtained by injecting AAV5.CaMKII.Cre-mCherry into the DG of *Grin2d*-floxed mice. Littermates injected with AAV5.CaMKII.mCherry served as controls. NMDAR EPSCs were recorded from mCherry-expressing GC and electrically evoked by LPP stimulation. Right, fluorescence image showing the viral expression in the DG of *Grin2d*-floxed mice. **(E)** Heterosynaptic LTP at LPP-GC synapses was abolished in GC *Grin2d* cKOs (*Grin2d* postsynaptic cKO: black circles, 97.8 ± 4.1%, *p* = 0.18, *n* = 4, 3 mice, paired *t*-test, g_*av*_ = –0.20). LTP was unaffected in control animals (white circles, 144.5 ± 11.2%, *p* = 0.02, *n* = 5, 4 mice, paired *t*-test, g_*av*_ = 3.84; cKO vs. control: *p* < 0.001, unpaired *t*-test, *g* = –8.35). Numbers in parentheses indicate the number of cells. Summary data represent mean ± s.e.m.

**FIGURE 5:**
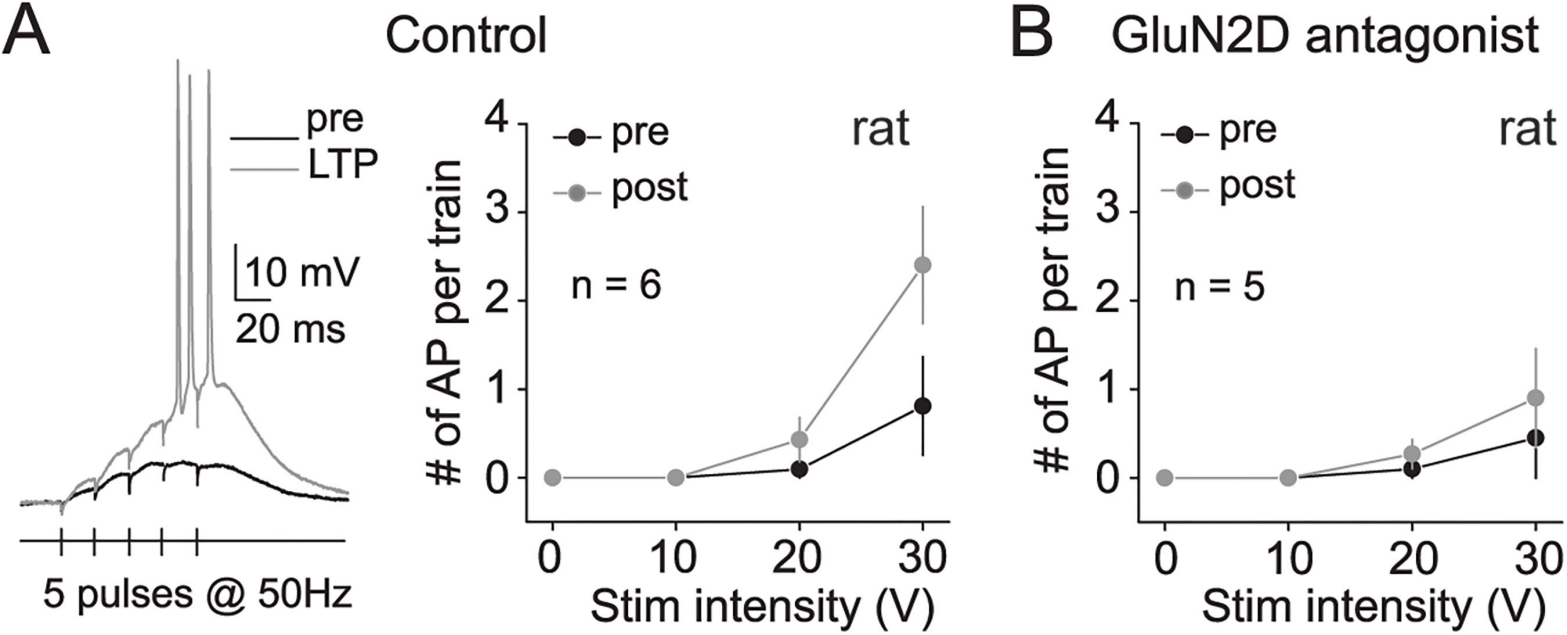
Induction of heterosynaptic NMDAR-LTP can increase GC output. **(A)** Left, traces of whole-cell current-clamp recordings in a GC in response to LPP stimulations (30 V), before (black trace) and after induction (gray trace) of heterosynaptic NMDAR LTP. NMDAR EPSPs were evoked using a train of stimulations (5 pulses at 50 Hz) of LPP inputs. Pre-post pairing LTP induction protocol was delivered at MPP inputs. Right, Average number of action potentials (APs) per train at different stimulation intensities (10, 20, and 30 V), showing how heterosynaptic induction of NMDAR LTP increases LPP-driven GC firing under control conditions [two-way ANOVA RM; pre vs. post LTP protocol: *F*(1, 6) = 20.0, *p* = 0.004; stimulation intensity: *F*(1.0, 6.2) = 10.2, *p* = 0.018; pre vs. post LTP protocol × stimulation intensity: *F*(1.2, 7.4) = 9.0, *p* = 0.016, *n* = 6, *N* = 4]. **(B)** Summary plot showing average number of APs per LPP stimulation train across stimulation intensities, before and after application of the LTP induction protocol, in the continuous presence of GluN2D antagonist [DQP-1105 (30 μM) or QNZ46 (30 μM)]. GluN2D antagonism prevented the increase in LPP-driven GC firing [two-way ANOVA RM; pre vs. post LTP protocol: *F*(1, 4) = 1.42, *p* = 0.30; stimulation intensity: *F*(1, 4) = 2.06, *p* = 0.22; pre vs. post LTP protocol × stimulation intensity: *F*(1, 4) = 1.3, *p* = 0.32, *n* = 5, *N* = 4].

To stimulate MPP and LPP inputs to GCs, stimulating patch-type pipettes were placed in the middle third of the medial molecular layer and in the distal part of the outer molecular layer of the dentate gyrus, respectively. To elicit synaptic responses, monopolar square-wave voltage or current pulses (100–200 μs pulse width, 4–30 V or 20–100 μA) were delivered through a stimulus isolator (Isoflex, AMPI, or Digitimer DS2A-MKII) connected to a broken tip (∼10–20 μm) stimulating patch-type micropipette filled with ACSF. Typically, stimulation was adjusted to obtain comparable-magnitude synaptic responses across experiments; e.g., 40–100 pA NMDAR-EPSCs [holding potential (Vh) = −45 mV, Figures 1–4]. Input/output experiments in Figure 5 show NMDAR excitatory postsynaptic potentials (NMDAR-EPSPs; Vh ∼−60 mV) evoked at stimulation intensities of 10, 20, and 30 V. Burst-timing NMDAR plasticity was induced in current clamp mode (Vh ∼−60 to −70 mV) by pairing presynaptic bursts (6 pulses at 50 Hz) with a postsynaptic burst of action potentials (5 action potentials at 100 Hz) delivered at a 10 ms interval, repeated 100 times at 2 Hz. Averaged traces include 20 consecutive individual responses.

### Postsynaptic *Grin2d* conditional knockout

*Grin2d ^*fl*/*fl*^* mice were injected with Cre-expressing (AAV5-CaMKII-Cre-mCherry, 5.8 × 10^12^ Virus Molecules/mL, UNC Vector) or control (AAV5-CaMKII-mCherry, 4.9 × 10^12^ Virus Molecules/mL, UNC Vector) adeno-associated viruses. 1 μL of virus was injected (flow rate of 0.1 μL/min) unilaterally into the DG (relative to bregma: 2.06 mm posterior, 1.5 mm lateral, 1.8 mm ventral) of 5–6-week-old *Grin2d ^*fl*/*fl*^* mice. Animals were placed in a stereotaxic frame and anesthetized with isoflurane (up to 5% for induction and 1%–2% for maintenance). Slices for electrophysiology were prepared from injected animals 3–4 weeks after injection.

### Data analysis

Electrophysiological data were acquired at 5 kHz, filtered at 2.4 kHz, and analyzed using custom IgorPro software (Wavemetrics Inc.). The magnitude of LTP was assessed by comparing 10-min baseline responses with those recorded 20–30 min after LTP induction. A minimum of three animals were used for each experimental group.

### Reagents

Reagents were bath-applied after dilution into ACSF from stock solutions stored at −20 °C and prepared in water or DMSO, depending on the manufacturer’s recommendations. The final DMSO concentration was <0.01%. Cyclopiazonic acid, heparin, ryanodine, DQP-1105, QNZ46, SCH 50911, D-APV, and MPEP were purchased from Tocris Biosciences. NBQX was purchased from Cayman Chemical Co, while BAPTA, picrotoxin, and all salts for making ACSF and internal solutions were purchased from Sigma-Millipore.

### Quantification and statistical analysis

Statistical analysis was performed using OriginPro software (OriginLab). Normality of distributions was assessed using the Shapiro–Wilk test. For normal distributions, unpaired and paired two-tailed Student’s *t*-tests were used to assess between-group and within-group differences, respectively. For non-normal distributions, the nonparametric paired-sample Wilcoxon signed-rank test and the Mann–Whitney U test were used. Statistical comparisons in Figure 5 were performed using two-way ANOVA with repeated measures (RM), and Greenhouse–Geisser was used for correction of degrees of freedom when sphericity was not assumed.

To compare changes in EPSC amplitudes between groups, effect sizes were calculated using Hedges’ g for independent samples, comparing control and experimental groups. Hedges’ g is a bias-corrected version of Cohen’s d that reduces the overestimation of effect sizes in small samples, a common limitation in electrophysiological studies. This approach is recommended for biomedical data integration and meta-analyses (Lakens, 2013).


$$d=\frac{M\,e\,a\,n\,1-M\,e\,a\,n\,2}{\sqrt{\frac{\left(n\,1-1\right)\,S\,D\,1^{2}+\left(n\,2-1\right)\,S\,D\,2^{2}}{n\,1+n\,2-2}}}$$

$$g=d*\left(1-\frac{3}{\left(4\,\left(n\,1+n\,2\right)-9\right)}\right)$$

To quantify changes in synaptic strength before and after application of LTP induction protocol, effect sizes were calculated using Hedges’ g for repeated-measures designs (g_*av*_). Uncorrected effect sizes were first computed using the average variance of the baseline and 20–30 min post-induction measurements, providing a standardized metric comparable across experiments and studies. To correct for the upward bias associated with small sample sizes, Cohen’s d_*av*_ was multiplied by a small-sample correction factor based on the paired-samples degrees of freedom (df = *n*−1) (Lakens, 2013).


da⁢v=M⁢e⁢a⁢np⁢o⁢s⁢t−M⁢e⁢a⁢np⁢r⁢eS⁢Dp⁢o⁢s⁢t2+S⁢Dp⁢r⁢e22

$$g_{a\,v}=d_{a\,v}*\left(1-\frac{3}{\left(4\,\left(n-1\right)-1\right)}\right)$$

Statistical significance was set at *p* < 0.05. All values are reported as the mean ± SEM.

## Results

### LPP-GC synapses express robust heterosynaptic NMDAR LTP but heterosynaptic AMPAR LTD

We examined whether inducing burst timing-dependent NMDAR-LTP at MPP-GC synapses could trigger plasticity at distal LPP-GC synapses. To test for NMDAR heterosynaptic plasticity, NMDAR excitatory postsynaptic currents (NMDAR-EPSCs) were monitored in the same GC in response to MPP and LPP stimulation with patch-type micropipettes placed in the MML and at the most distal border of the outer molecular layer (OML) of the dentate gyrus, respectively (see “Materials and methods”). Both pipettes were positioned on the same side of the GC to activate similar dendritic branches (Figures 1A, B). To isolate NMDAR-mediated transmission, whole-cell patch-clamp recordings of GCs [holding potential (Vh) = −45 mV] were performed in continuous presence of 10 μM NBQX and 100 μM picrotoxin to block AMPAR- and GABA_*A*_ receptor-mediated transmission, respectively. After a 10-min baseline, we applied a burst pairing protocol (Harnett et al., 2009, Hunt et al., 2013) known to induce homosynaptic NMDAR-LTP at MPP-GC synapses (Berthoux et al., 2026), consisting of presynaptic stimulation (6 pulses at 50 Hz, at MPP) paired with postsynaptic firing (5 pulses at 100 Hz) with a 10 ms interval between pre- and postsynaptic activity, repeated 100 times (see “Materials and methods”). While homosynaptic AMPAR-LTP often induces compensatory, heterosynaptic AMPAR-LTD (Chater and Goda, 2021, Malyshev et al., 2026, Jenks et al., 2021), we found that homosynaptic MPP-GC NMDAR-LTP was accompanied by heterosynaptic NMDAR-LTP at LPP-GC synapses (Figures 1A, B). No significant changes in input resistance were observed after LTP induction (97.37% ± 3.25% of baseline, *n* = 11, *p* = 0.44, paired *t*-test), supporting the stability of the recordings. We further confirmed that both homosynaptic and heterosynaptic NMDAR-LTP can also be elicited at a more physiological temperature (32 ± 1°C) (Supplementary Figure 1). Remarkably, applying the pre-post burst stimulation protocol only to LPP-GC synapses did not trigger any form of homosynaptic or heterosynaptic NMDAR long-term plasticity (Figure 1C), indicating that heterosynaptic NMDAR-LTP was not caused by stimulation crosstalk between the MPP and LPP inputs.

Because single dendritic branches perform local computations and may act as fundamental functional units of a neuron (Kastellakis et al., 2023, Branco and Häusser, 2010), we hypothesized that the postsynaptic signaling that mediates NMDAR heterosynaptic plasticity could be restricted to the dendritic branches expressing homosynaptic LTP. To test this possibility, we placed the LPP stimulation pipette on the opposite side of the recorded GC (at least 250 μm lateral from the MPP stimulation pipette), which is expected to activate LPP inputs onto different dendritic branches. We observed that these LPP inputs did not exhibit heterosynaptic plasticity (Figure 1D), supporting the idea that NMDAR heterosynaptic plasticity depends on signaling confined to dendritic branches where homosynaptic LTP was induced. Lastly, we tested whether heterosynaptic LPP-GC NMDAR-LTP could also be observed in mice and repeated the experiment described in Figures 1A, B in acute mouse hippocampal slices. Both homosynaptic and heterosynaptic NMDAR plasticity were present in mice (Figure 1E), indicating a conserved plasticity mechanism. Overall, these results demonstrate that MPP and LPP inputs onto GCs undergo distinct forms of NMDAR plasticity. While pre-post burst stimulation induced homosynaptic NMDAR-LTP at MPP inputs (Berthoux et al., 2026), only heterosynaptic NMDAR-LTP was observed at LPP inputs.

We wondered whether the homosynaptic and heterosynaptic BTDP we observed were exclusive to NMDARs or if they could also be exhibited by AMPARs. While almost nothing is known about NMDAR heterosynaptic plasticity, several reports have indicated that AMPARs can undergo heterosynaptic changes in neighboring synapses (Chater and Goda, 2021, Malyshev et al., 2026, Jenks et al., 2021). However, only a few *in vivo* studies, with mixed results, have explored heterosynaptic AMPAR plasticity in the dentate gyrus (Abraham et al., 2007, Bromer et al., 2018, Jungenitz et al., 2018), and to our knowledge, no *in vitro* study has characterized any form of functional heterosynaptic AMPAR plasticity at LPP-GC synapses. To fill this knowledge gap, we monitored AMPAR-EPSCs at both MPP- and LPP-GC synapses within the same GC (Vh = −60 mV), in the presence of the GABA_*A*_ receptor antagonist picrotoxin (100 μM). After establishing a baseline, we applied pre-post burst stimulation at MPP-GC synapses and observed homosynaptic AMPAR-LTP at MPP-GC synapses but heterosynaptic AMPAR-LTD at LPP-GC synapses (Figure 1F). These findings indicate that BTDP modifies the relative contribution of AMPA and NMDA receptors at MPP and LPP synaptic inputs.

### Internal Ca^2+^ stores are required for heterosynaptic but not homosynaptic NMDAR-LTP

NMDAR-LTP at central nervous system synapses (Hunt and Castillo, 2012), including MPP-GC synapses (Harney et al., 2006, O’Connor et al., 1994), relies on a postsynaptic induction mechanism involving NMDAR and type 5 metabotropic glutamate receptor (mGluR5) co-activation and a rise in postsynaptic [Ca^2+^]. We found that bath application of the mGluR5 antagonist MPEP (4 μM) (Figures 2A, B) and intracellular loading of the Ca^2+^ chelating agent BAPTA (10 mM) abolished LTP (Figure 2C), indicating that both mGluR5 and postsynaptic Ca^2+^ are required for pre-post burst-induced NMDAR LTP at MPP-GC synapses. Additionally, these manipulations also prevented heterosynaptic NMDAR-LTP at LPP-GC synapses recorded from the same GC (Figures 2D, E), suggesting a shared induction mechanism.

Internal Ca^2+^ stores contribute to the expression of homosynaptic NMDAR-LTP at mossy fiber to CA3 pyramidal cell synapses (Hunt et al., 2013). We then tested the role of internal Ca^2+^ stores in both homosynaptic and heterosynaptic NMDAR-LTP at dentate GC synapses. To this end, we used the cell-permeable inhibitor of sarcoplasmic reticulum Ca^2+^-ATPase, cyclopiazonic acid (CPA 30 μM, 40–50 min pre-incubation, also included in the perfusion) to deplete internal Ca^2+^ stores (Figures 3A, B). We found that CPA abolished the induction of LPP-GC heterosynaptic plasticity (Figure 3A) but had no effect on MPP-GC homosynaptic plasticity (Figure 3B). Internal Ca^2+^ can be released from endoplasmic reticulum (ER) stores via IP3 signaling or ryanodine receptor (RyR) activation. To determine which of these mechanisms is involved in heterosynaptic NMDAR-LTP, we incubated (40–50 min) and perfused hippocampal slices with heparin (2 mg/ml) and ryanodine (100 μM) to block IP3-mediated and RyR-mediated Ca^2+^ release, respectively (Figures 3C, D). We found that induction of heterosynaptic LPP-GC NMDAR-LTP was abolished by ryanodine but normally induced in the presence of heparin (Figure 3C), indicating that heterosynaptic LTP requires RyRs but not IP3 receptors. In contrast, homosynaptic NMDAR-LTP at MPP-GC was normally induced in the presence of both ryanodine and heparin (Figure 3D), strongly suggesting that the rise in postsynaptic Ca^2+^ at MPP-GC synapses required for this form of plasticity is independent of internal Ca^2+^ stores. Collectively, these findings demonstrate that homosynaptic vs. heterosynaptic NMDAR-LTP have distinct Ca^2+^ requirements for induction, with internal Ca^2+^ release necessary only for heterosynaptic NMDAR-LTP at LPP-GC synapses.

### Heterosynaptic NMDAR-LTP is likely mediated by the synaptic recruitment of GluN2D-containing receptors

Previous studies reported that homosynaptic NMDAR-LTP at MPP-GC synapses is mediated by the recruitment of GluN2D-containing NMDARs to the synapse (Harney et al., 2008, Berthoux et al., 2026). To assess the role of GluN2D subunits in burst timing-dependent heterosynaptic NMDAR-LTP, we first used two different non-competitive, activity-dependent GluN2C/GluN2D selective antagonists, DQP-1105 and QNZ46 (Hansen and Traynelis, 2011, Monaghan et al., 2012). Although these antagonists may not distinguish between GluN2C- and GluN2D-containing NMDARs, GluN2C expression in the adult hippocampus (Sanz-Clemente et al., 2013) and forebrain neurons (Alsaad et al., 2019) is negligible. We first tested the effect of DQP-1105 on heterosynaptic LPP-GC NMDAR-LTP and found that bath application of 30 μM DQP-1105 abolished heterosynaptic plasticity (Figure 4A). This manipulation also blocks homosynaptic NMDAR-LTP at MPP-GC synapses (Berthoux et al., 2026). Most evidence indicates that GluN2D-containing receptors are primarily extrasynaptic in adult animals (Paoletti et al., 2013). Consistent with this, bath application of the antagonists DQP-1105 or QNZ46 (30 μM) had no effect on LPP-GC basal synaptic transmission, whereas the non-selective NMDAR antagonist D-APV (50 μM) abolished these responses (Figure 4B). Remarkably, application of the selective GluN2D antagonists (DQP-1105 or QNZ46, 30 μM) 30 min after the induction of NMDAR-LTP significantly reduced LPP-GC NMDAR-mediated transmission (Figure 4C). These data strongly suggest that, like homosynaptic NMDAR-LTP, heterosynaptic NMDAR-LTP in the dentate gyrus likely results from the recruitment of GluN2D-containing receptors to the synapse.

To demonstrate the role of postsynaptic GluN2D-containing NMDARs in heterosynaptic NMDAR-LTP, we employed a conditional knockout strategy to selectively remove *Grin2d* from GCs (*Grin2d* postsynaptic cKO) by stereotaxically injecting adeno-associated virus (AAV) expressing Cre recombinase (AAV5.CaMKII.Cre-mCherry) or mCherry alone (AAV5.CaMKII.mCherry) into the dentate gyrus of adult *Grin2d*-floxed mice (Figure 4D). We recorded from mCherry^+^ GCs and found that heterosynaptic NMDAR-LTP was abolished in Cre-mCherry^+^ GCs (*Grin2d* postsynaptic cKO), whereas it remained intact in mCherry^+^ GCs (control) (Figures 4D, E). Consistent with findings that *Grin2d* postsynaptic cKO prevents homosynaptic NMDAR potentiation at MPP-GC synapses, these results suggest that postsynaptic GluN2D-containing NMDARs are also necessary for heterosynaptic NMDAR-LTP at LPP-GC synapses.

### Heterosynaptic NMDAR-LTP enhances synaptically-driven GC output

We next sought to determine the functional impact of heterosynaptic NMDAR-LTP and whether it enhances the ability of LPP inputs to drive action potential (AP) firing in dentate GCs. To address this, we performed whole-cell current-clamp recordings from GCs and measured the probability of AP firing in response to LPP burst stimulation, which consisted of five pulses delivered at 50 Hz with varying stimulus strengths. NMDAR excitatory postsynaptic potentials (NMDAR-EPSPs) were recorded in the presence of NBQX (10 μM), picrotoxin (100 μM), and SCH50911 (20 μM) to block AMPA/Kainate and GABA_*A*_ and GABA_*B*_ receptors, respectively. We monitored LPP-evoked burst firing before and 15–20 min after LTP induction. Our results showed that inducing heterosynaptic NMDAR-LTP was associated with a significant increase in the number of APs in GCs (Figure 5A). No significant changes were observed in membrane input resistance post-LTP induction (R_*input*_, pre-LTP: 249.1 ± 41.7 MΩ, post-LTP: 230.7 ± 52.6 MΩ, *n* = 7, *p* = 0.26, paired *t*-test; data not shown). To test whether the increase in firing was mediated by GluN2D-dependent plasticity, we repeated the experiment in the presence of the selective GluN2D antagonist DQP-1105 (30 μM), which abolished heterosynaptic NMDAR-LTP at LPP-GC synapses (Figure 4A). Under these conditions, application of the pre–post burst stimulation protocol at MPP–GC synapses did not produce a significant change in LPP-evoked AP firing (Figure 5B). Together, these findings strongly suggest that GluN2D-mediated heterosynaptic NMDAR LTP enhances GC output driven by LPP burst activity.

## Discussion

In this study, we describe a form of activity-dependent plasticity at entorhinal-dentate granule cell synapses, characterized by heterosynaptic potentiation of NMDAR-mediated transmission. Using a physiologically relevant burst timing-dependent paradigm, we show that triggering homosynaptic NMDAR-LTP at MPP synapses also causes robust heterosynaptic NMDAR-LTP at distal, non-stimulated LPP synapses. In contrast, the same induction protocol that triggered homo- and heterosynaptic NMDAR-LTP induced homosynaptic AMPAR-LTP at MPP-GC synapses but heterosynaptic AMPAR-LTD at LPP-GC synapses. Heterosynaptic NMDAR-LTP depended on Ca^2+^ release from internal stores, likely implicating long-range signaling via the ER. Additionally, the increased sensitivity to GluN2D antagonists after the induction of both homosynaptic and heterosynaptic NMDAR-LTP suggests that these forms of plasticity involve the synaptic recruitment of GluN2D-containing NMDARs. NMDAR-mediated synaptic strengthening results in a sustained increase in synaptically-driven GC activity. Together, our findings revealed a new activity-dependent synaptic mechanism in which functionally distinct entorhinal inputs work together to control the output of the dentate gyrus.

### MPP-GC but not LPP-GC synapses exhibit homosynaptic NMDAR-LTP

BTDP of NMDAR-mediated transmission has previously been reported in midbrain dopamine neurons (Harnett et al., 2009), GC-CA3 synapses (Hunt et al., 2013), and MPP-GC synapses (Berthoux et al., 2026). *In vivo* recordings showed that neurons from the medial entorhinal cortex fire bursts of action potentials (Csordás et al., 2020, Ebbesen et al., 2016, Latuske et al., 2015), and GCs, which normally fire sparsely, can generate high-frequency bursts of back-propagating action potentials (Diamantaki et al., 2016, Pernía-Andrade and Jonas, 2014, Senzai and Buzsáki, 2017). In the present study, we found that pairing pre and postsynaptic burst activity, which selectively induces homosynaptic NMDAR-LTP at MPP-GC (Berthoux et al., 2026), did not induce homosynaptic plasticity at LPP-GC synapses under our experimental conditions. This finding might be explained by a strong dendritic attenuation of backpropagating action potentials as a function of distance from the soma (Kim et al., 2018, Krueppel et al., 2011). Also, as in distal synapses of other pyramidal neurons (Larkum et al., 2001, Sjöström and Nelson, 2002), backpropagating action potentials may not be sufficient to unblock NMDARs at LPP-GCs. In a recent study, a more standard pre-post pairing protocol (i.e., single action potentials and synaptic responses) also failed to elicit LPP-GC AMPAR plasticity, whereas this plasticity was induced by theta-burst stimulation of LPP inputs that generated local dendritic spikes (Kim et al., 2018). Our findings on NMDAR-mediated transmission support the idea that synaptic plasticity rules at proximal and distal entorhinal inputs onto GCs differ.

### MPP-GC burst activity triggers opposite heterosynaptic LPP-GC plasticity of NMDAR- and AMPAR-mediated transmission

In this study, we present evidence of heterosynaptic NMDAR plasticity. Due to the unique properties of NMDARs, which can cause signal amplification, temporal summation, and increased Ca^2+^ influx (Hunt and Castillo, 2012), it is likely that NMDAR potentiation modifies the dendritic properties of GCs at distal LPP inputs during heterosynaptic NMDAR LTP. Unlike NMDAR-mediated transmission, AMPAR potentiation at MPP-GC synapses was accompanied by heterosynaptic AMPAR-LTD at LPP-GC synapses. Since its initial discovery in the CA1 area (Lynch et al., 1977), similar heterosynaptic LTD of AMPAR-mediated transmission has been reported at several other synapses (Chater and Goda, 2021, Malyshev et al., 2026, Jenks et al., 2021). Our findings provide further evidence that NMDARs and AMPARs exhibit opposite heterosynaptic plasticity rules, which might affect dendritic integration in GCs. While heterosynaptic NMDAR-LTP may enhance information transfer from LPP inputs to GCs, likely by facilitating temporal summation (Drotos et al., 2025), heterosynaptic AMPAR-LTD (Figure 1F) is expected to reduce the LPP-mediated drive of GCs. Therefore, concurrent induction of both forms of BTDP might favor burst-only LPP inputs. The potential role of such winner-take-all dynamics in the dentate gyrus warrants further exploration.

### Homosynaptic vs. heterosynaptic NMDAR LTP have unique Ca^2+^ requirements

Mechanistically, the induction and expression of NMDAR plasticity share common properties across synapses (Hunt and Castillo, 2012, Rebola et al., 2010), including postsynaptic NMDAR-mediated Ca^2+^ influx and Ca^2+^ release from internal stores. A pivotal finding of our study is the mechanistic divergence between homosynaptic and heterosynaptic NMDAR plasticity. While homosynaptic MPP-GC NMDAR-LTP is unaffected by the depletion of internal Ca^2+^ stores, heterosynaptic potentiation at LPP synapses is abolished by ryanodine. Although ryanodine exhibits a complex, biphasic dose-response profile, the concentration used selectively blocks intracellular calcium release (Wang et al., 1996). However, we cannot rule out potential off-target effects. This dependence on RyR-mediated Ca^2+^ release suggests a long-range signaling mechanism that compensates for the dendritic attenuation of back-propagating action potentials in GCs (Kim et al., 2018, Krueppel et al., 2011). Homosynaptic NMDAR-LTP may alter the synaptic rules at distal LPP-GC synapses, by eliciting the release of internal ER Ca^2+^ stores, although the diffusion of other signaling molecules cannot be discarded (Chater and Goda, 2021, Jenks et al., 2021). While homosynaptic NMDAR-LTP at GC-CA3 synapses requires IP3-dependent Ca^2+^ release (Hunt et al., 2013, Kwon and Castillo, 2008), evidence in the amygdala shows that heterosynaptic, but not homosynaptic plasticity, requires RyR-dependent Ca^2+^-induced Ca^2+^ release from internal stores (Royer and Paré, 2003). A role for NMDAR-dependent Ca2^+^-induced Ca^2+^ release from the ER in heterosynaptic plasticity of AMPAR-mediated transmission has also been demonstrated in CA1 pyramidal neurons (Nishiyama et al., 2000, Oh et al., 2015, Lee et al., 2016) and neocortex (Field et al., 2020). Furthermore, in mesolimbic dopamine neurons exposed to alcohol, increased susceptibility to induce NMDAR-LTP relies on Ca^2+^ release from ER stores (Bernier et al., 2011). Electron microscopy studies of pyramidal hippocampal neurons show that the smooth ER extends into distal dendrites and dendritic spines (Spacek and Harris, 1997). The coincident increase in ER-Ca^2+^ sources, along with increased GluN2D recruitment to potentially ease Mg^2+^ block, might provide an ideal scenario for the induction and expression of heterosynaptic NMDAR LTP at distal synapses. Future *in vivo* Ca^2+^ imaging studies combined with genetic strategies could clarify how these two events converge to mediate heterosynaptic plasticity and potentially contribute to dentate gyrus-dependent learning.

### Heterosynaptic NMDAR-LTP is mediated by GluN2D recruitment

GluN2D expression in the adult hippocampus is generally believed to be primarily restricted to interneurons (Dubois and Liu, 2021, Monyer et al., 1994, Perszyk et al., 2016, von Engelhardt et al., 2015). However, GluN2D is robustly expressed in the dendrites and axons of adult glutamatergic excitatory neurons in the subthalamic nucleus (Swanger et al., 2015), and GluN2D-containing NMDARs have also been reported in rat postnatal dentate gyrus GCs (Piña-Crespo and Gibb, 2002). As for homosynaptic MPP-GC synapses (Harney et al., 2008, Berthoux et al., 2026), the maintenance of heterosynaptic LPP-GC NMDAR-LTP was also significantly reduced by both GluN2D-subunit antagonists and postsynaptic *Grin2d* cKO, suggesting that both forms of plasticity require recruitment of GluN2D-containing receptors to the synapse, presumably forming triheteromers (GluN1/2B/2D) or diheteromers (GluN1/2D) (Dunah et al., 1998, Hansen et al., 2017, Paoletti et al., 2013, Kang et al., 2025). GluN1/2B/2D triheteromers have functional properties intermediate to those of GluN1/2B and GluN1/2D diheteromers (Yi et al., 2019). The structural identification of functional triheteromeric GluN1-2B-2D receptors in the adult brain (Kang et al., 2025) confirms that GluN2D is a stable and functional component of the mature NMDAR landscape. While the recruitment of GluN1/2D diheteromers could result in NMDAR-EPSCs with a slower decay (Cull-Candy et al., 2001, Hansen and Traynelis, 2011, Sanz-Clemente et al., 2013), no differences in NMDAR-EPSC decay were observed before and after homosynaptic LTP (Harney et al., 2008), suggesting that GluN2D-containing NMDARs are primarily triheteromers. Importantly, a key property of GluN1/2D diheteromers is the low sensitivity to Mg^2+^ blockade relative to GluN2A or GluN2B-containing receptors (Paoletti et al., 2013). Although backpropagating APs at distal synapses may not be sufficient to remove the NMDAR block (Larkum et al., 2001, Sjöström and Nelson, 2002), expression of GluN2D-containing NMDARs after NMDAR LTP induction may be one factor that helps explain why NMDARs exhibit different synaptic plasticity learning rules than AMPARs.

By increasing the synaptically driven activity of GCs (Figure 5), heterosynaptic NMDAR-LTP could facilitate the transfer of information from the LPP to the dentate GCs, thereby dynamically regulating dentate gyrus computations and memory processes. Consistent with this idea, GC *Grin2d* cKO, which abolished heterosynaptic NMDAR LTP (Figure 4D), can also impair DG-dependent forms of memory (Berthoux et al., 2026). Our finding that GluN2D antagonists can block the LPP-evoked GC firing after NMDAR-LTP induction aligns with previous work showing that spike firing rate was increased by the GluN2C/D-positive allosteric modulator CIQ, and decreased by the GluN2C/D antagonist DQP-1105 in subthalamic neurons *in vivo* (Swanger et al., 2015). The low sensitivity of GluN2D to voltage-dependent Mg^2+^ block allows these receptors to pass current even at or near resting membrane potentials (Clarke and Johnson, 2006, Qian et al., 2005). Notably, heterosynaptic changes in NMDAR subunit composition have been previously reported in CA1 neurons (Han and Heinemann, 2013). GluN2D-mediated synaptic transmission has been reported in hippocampal interneurons of adult mice (von Engelhardt et al., 2015, Perszyk et al., 2016, Yi et al., 2019), but it remains unclear whether activity modulates the expression of synaptic GluN2D-containing NMDARs in these neurons.

A potential limitation of the present study is that sex-specific differences in NMDAR heterosynaptic plasticity were not fully addressed. Although data from male and female subjects were pooled for analysis because no major sex differences were observed, we cannot exclude the possibility of more subtle sexually dimorphic effects. Furthermore, fluctuations in ovarian hormones during the estrous cycle may influence synaptic plasticity. Future studies with larger sample sizes and systematic assessment of estrous cycle stage will be required to provide sufficient statistical power to detect potential sex-dependent differences and to determine whether the mechanisms underlying NMDAR-dependent heterosynaptic plasticity differ between males and females.

In summary, BTDP of NMDAR-mediated transmission at MPP synapses can exert long-term, powerful control over NMDAR transmission at distal LPP-GC synapses. The coexistence of NMDAR heterosynaptic LTP and AMPAR heterosynaptic LTD at distal LPP synapses suggests a sophisticated regulatory mechanism for GC integration. Dynamic changes in NMDAR transmission could shift the induction threshold of NMDAR-dependent forms of plasticity, as seen in CA3 neurons, where NMDARs can selectively adjust synapses in a heterosynaptic manner (Tsukamoto et al., 2003), serve as a metaplastic switch for AMPAR-mediated plasticity (Astori et al., 2010, Rebola et al., 2011), and contribute to the metaplasticity of AMPAR LTP (Hunt et al., 2013). Behavioral deficits in GC *Grin2d* cKO mice (Berthoux et al., 2026) suggest a potential role for homo- and heterosynaptic plasticity in hippocampal-dependent behavior, but further work is required to determine the precise contribution of homo- and heterosynaptic NMDAR plasticity in behaving animals.

## Data Availability

The raw data supporting the conclusions of this article will be made available by the authors, without undue reservation.
